# The correlation of epithelial-mesenchymal transition-related gene expression and the clinicopathologic features of colorectal cancer patients in Taiwan

**DOI:** 10.1371/journal.pone.0254000

**Published:** 2021-07-02

**Authors:** Yuan-Chang Dai, Chuan-Yin Fang, Hsin-Yi Yang, Yi-Jun Jian, Shou-Chieh Wang, Yi-Wen Liu

**Affiliations:** 1 Department of Microbiology, Immunology and Biopharmaceuticals, College of Life Sciences, National Chiayi University, Chiayi City, Taiwan; 2 Department of Pathology, Ditmanson Medical Foundation Chiayi Christian Hospital, Chiayi City, Taiwan; 3 Department of Laboratory Medicine, Ditmanson Medical Foundation Chiayi Christian Hospital, Chiayi City, Taiwan; 4 Department of Colorectal Surgery, Ditmanson Medical Foundation Chiayi Christian Hospital, Chiayi City, Taiwan; 5 Clinical Medicine Research Center, Ditmanson Medical Foundation Chiayi Christian Hospital, Chiayi City, Taiwan; 6 Division of Nephrology, Department of Internal Medicine, Kuang Tien General Hospital, Taichung, Taiwan; Aix-Marseille University, FRANCE

## Abstract

Colorectal cancer (CRC) is the third most commonly diagnosed malignancy and the second leading cause of cancer-related deaths in the world. It has been the most prevalent malignancy in Taiwan for consecutive thirteen years. Despite the diversity of its etiologic and pathophysiologic factors, a biological process named as epithelial-mesenchymal transition (EMT) is indispensable in the progression of epithelial cancer. Our aim is to investigate the correlation between the expression of 8 EMT-related proteins (E-cadherin, β-catenin, claudin-1, CD44, N-cadherin, fibronectin, vimentin, S100A4) and the clinicopathologic features of CRC in Taiwan, along with the DNA CpG epigenetic status of *CD44* gene. In immunohistochemical assessment, decreased expression of E-cadherin is statistically associated with the progression of cancer stage, while decreased expression of claudin-1 as well as increased β-catenin nuclear translocation and N-cadherin expression is statistically associated with the progression of histopathologic grade. E-cadherin, nuclear β-catenin and claudin-1 are also associated with other important prognostic factors, including nodal metastasis, tumor deposits, and elevated serum CA 19–9 levels. In addition, the left-sided colon and rectal cancers show increased nuclear translocation of β-catenin compared to the right-sided colon cancers, while the rectal cancers show increased fibronectin expression compared to the right-sided and left-sided colon cancers. Moreover, vimentin is aberrantly expressed in one case of signet-ring cell carcinoma. The DNA methylation levels of *CD44* gene promoter between the tumoral and non-tumorous tissues by NGS comparison showed statistical difference on six CpG sites. However, such difference may not be sufficient because these DNA methylation proportions are too low to inactivate *CD44* gene. Our results demonstrate the expression of E-cadherin, claudin-1, and nuclear β-catenin is closely related to the clinicopathologic prognostic determinants of CRC in Taiwan. The DNA methylation level of *CD44* gene and its protein expression, however, show no correlation with the clinicopathologic features in CRC.

## Introduction

Colorectal cancer (CRC) is the third most commonly diagnosed malignancy and the second leading cause of cancer-related deaths in the world. It accounts for approximately 10% of all annually diagnosed cancers and cancer-related deaths worldwide [1]. In Taiwan, CRC occurs in 14.23% of all malignancies and causes 11.94% of cancer-related deaths. The incidence rate of CRC among all malignancies in Taiwan ranks 1st in male and 3rd in female; its cancer-related death rate ranks 3rd in male and 4th in female [2, 3].

Both hereditary and environmental risk factors play a part in the development of colorectal cancer [4]. Studies of colorectal carcinogenesis have provided fundamental insights into the general mechanisms of cancer evolution. The combination of molecular events that lead to colonic adenocarcinoma is heterogeneous and includes genetic and epigenetic abnormalities [4].

Although the diversity of the etiologic and pathophysiologic factors of CRC is very wide, one biological process named as epithelial-mesenchymal transition (EMT) is indispensable in the progression of these conditions [5]. An EMT is a biologic process that allows a polarized epithelial cell, which normally interacts with basement membrane via its basal surface, to undergo multiple biochemical changes that enable it to assume a mesenchymal cell phenotype, which includes enhanced migratory capacity, invasiveness, elevated resistance to apoptosis, and greatly increased production of extracellular matrix components [6]. The field of oncology has seen a recent explosion of EMT-related research for both prognostication and treatment of metastatic cancers, and to date, numerous classical EMT markers have been significantly correlated with metastasis. Moreover, recent works suggest that assessing classical markers of EMT may help clinicians predict resistance to chemotherapy, and thus poor prognosis [7].

For example, loss of CDH1 (E-cadherin) expression in CRC is associated with infiltrative tumor growth pattern and lymph node metastasis [8]. Vimentin expression increased in accordance with tumor progression of CRC, and the tumors with high expression of vimentin had a greater extent of tumor invasion to the serosa, lymph node metastasis and liver metastasis than those with low expression of vimentin [9]. In addition, DNA methylation of some EMT-related genes is associated with progression and prognosis of CRC. *CDH1* promoter hypermethylation was associated with later disease stage [10]. In CRC with methylation of *VIM* (vimentin gene), a trend was shown toward preferentially developing liver metastasis and peritoneal dissemination [11]. *CD44* has been reported that DNA hypermethylation of its promoter causes gene silence in prostate cancer [12, 13] and in gastric cancer cells [14]. DNA methylation is usually associated with the silencing of gene expression. However, a meta-analysis in CRC indicates that CD44 overexpression is not a good prognostic factor [15].

In summary, improving our understanding in the status of EMT and its regulation in CRC is of crucial importance, and could provide novel opportunities in the treatment of CRC patients by preventing cancer progression. In this study, for understanding the correlation of EMT-related protein expression and the clinicopathologic features of CRC in Taiwan, we analyzed the expression of 8 EMT-related proteins (E-cadherin, β-catenin, claudin-1, CD44, N-cadherin, fibronectin, vimentin, S100A4) by immunohistochemistry assay. Moreover, because the role of CD44 in the metastasis of CRC is still not completely understood and its DNA methylation status was not well studied, the DNA methylation of CD44 and the relationship of its protein expression with the clinicopathologic features of CRC were simultaneously analyzed.

The aim of our study is to investigate the correlation of the expression of 8 EMT-related proteins with the clinicopathologic features, and the DNA methylation status of *CD44* gene in CRC of Taiwan, which may in turn provide the information for prognosis evaluation and treatment prediction as well as the potentials of these markers as therapeutic targets.

## Materials and methods

### Clinical sample collection

Formalin-fixed and paraffin-embedded (FFPE) tumoral tissue specimens of 150 patients with CRC as well as 30 paired freshly frozen tumoral and adjacent non-tumorous mucosal tissue samples with 20 additional freshly frozen tumoral tissue samples, including the delinked clinicopathologic data, were obtained from the Biobank of Ditmanson Medical Foundation Chiayi Christian Hospital. All the donor patients have signed informed consents before they provided biospecimens to the Biobank of Ditmanson Medical Foundation Chia-Yi Christian Hospital. The informed consent documents were then stored in the Biobank. When the tissue specimens and the clinicopathologic data were requested from the Biobank for this research, the identity of the donor patients had been delinked. All the specimens and data were fully anonymized and recoded. All processes and researches were approved by the Institutional Review Board of Ditmanson Medical Foundation Chiayi Christian Hospital (Approval No. CYCH-IRB 106091). The tissue slides and the clinicopathologic data were obtained from the Biobank in October, 2018 and the frozen tissue specimens and the clinicopathologic data were obtained in March, 2019. The participated patients underwent colorectal surgery within the duration from March, 2016 to January, 2019.

### Immunohistochemistry (IHC)

Expression of eight EMT-related proteins (E-cadherin, β-catenin, claudin-1, CD44, N-cadherin, fibronectin, vimentin, S100A4) were explored by IHC and its correlation with the clinicopathologic features was assessed by statistical analysis.

The primary antibodies for EMT-related proteins, anti-E-cadherin (NCH-38, Dako Agilent Technologies, USA), anti-β-catenin (EP35, Zeta Corporation, USA), anti-claudin-1 (SP128, Zytomed System, German), anti-CD44 (SP37, Zeta Corporation, USA), anti-N-cadherin (IAR06, Leica Biosystem, German), anti-fibronectin (IST-9, Abcam, UK), anti-vimentin (V9, Leica Biosystem, German), and anti-S100A4 (EPR2761(2), Abcam, UK), were purchased from Bond Biotech Inc. (Tai-Chung, Taiwan). The immunohistochemical staining of FFPE tumoral tissue specimens was performed by BOND-III fully automated IHC and ISH stainer (Leica Biosystem, German).

### Genomic DNA extraction and bisulfite conversion

The genomic DNA of thirty paired freshly frozen tumoral and adjacent non-tumorous mucosal tissue samples as well as twenty additional freshly frozen tumoral tissue samples were extracted by Geno Plus genomic DNA miniprep system (Viogene, Taiwan) following the kit instructions. After checking the genomic DNA quality by a spectrophotometer and electrophoresis, five hundred nanograms of genomic DNA was reacted with sodium bisulfite following the EZ DNA methylation-Gold kit (Zymo Research, USA). After elution, the bisulfited products were stored at -80°C before use and for no more than 2 weeks.

### DNA amplification and purification

According to the information on NCBI-Gene website (https://www.ncbi.nlm.nih.gov/gene), there are 72% CpG sites among -500 bp to +129 bp of *CD44* transcriptional start site (TSS), which was analyzed by CpG island Finder program (http://dbcat.cgm.ntu.edu.tw/). Therefore, the bisulfite-specific PCR primers were designed as: forward primer 5’-GAATTTAGYGGGAAAGGAGAGGTTAAAGG-3’, and reverse primer 5’-AACCRAACCTAACAAAAACTAAAATCC-3’, which amplified the sequence -394 to +43 of human *CD44* gene. The bisulfite-specific PCR primers amplified the sequence of bisulfited products by Hot-start GoTaq DNA polymerase (Promega, USA). The amplified products were analyzed by electrophoresis, and the 437 bp bands were then purified by Gene-Spin 1-4-3 DNA purification kit (Protech Technology, Taiwan).

### Next-Generation Sequencing (NGS)

The purified PCR products were sequenced by NGS. After barcoding PCR, the products were purified and pooling mixed. The DNA products were constructed into library by Celero DNA-Seq kit (NuGEN, USA), then sequenced by Illumina MiSeq system (Illumina, USA). After trimming the raw data by CLC Genomics Workbench v10 (Qiagen, German), the sequences were analyzed to distinguish the methylated (C) or unmethylated (T) of total 27 CpG sites of *CD44* gene promoter. Each site was read over 30000 reads.

### Statistical analysis

Numerical data are expressed as the mean ± standard error for all samples. Statistical differences were analyzed by Chi-square test and *t*-test. *P* < 0.05 was considered significant and labeled as *. All statistics were calculated using SPSS Statistics version 21.0 (IBM, USA).

## Results

### The correction of IHC expression of EMT-related markers and histopathologic grades

**Fig 1:**
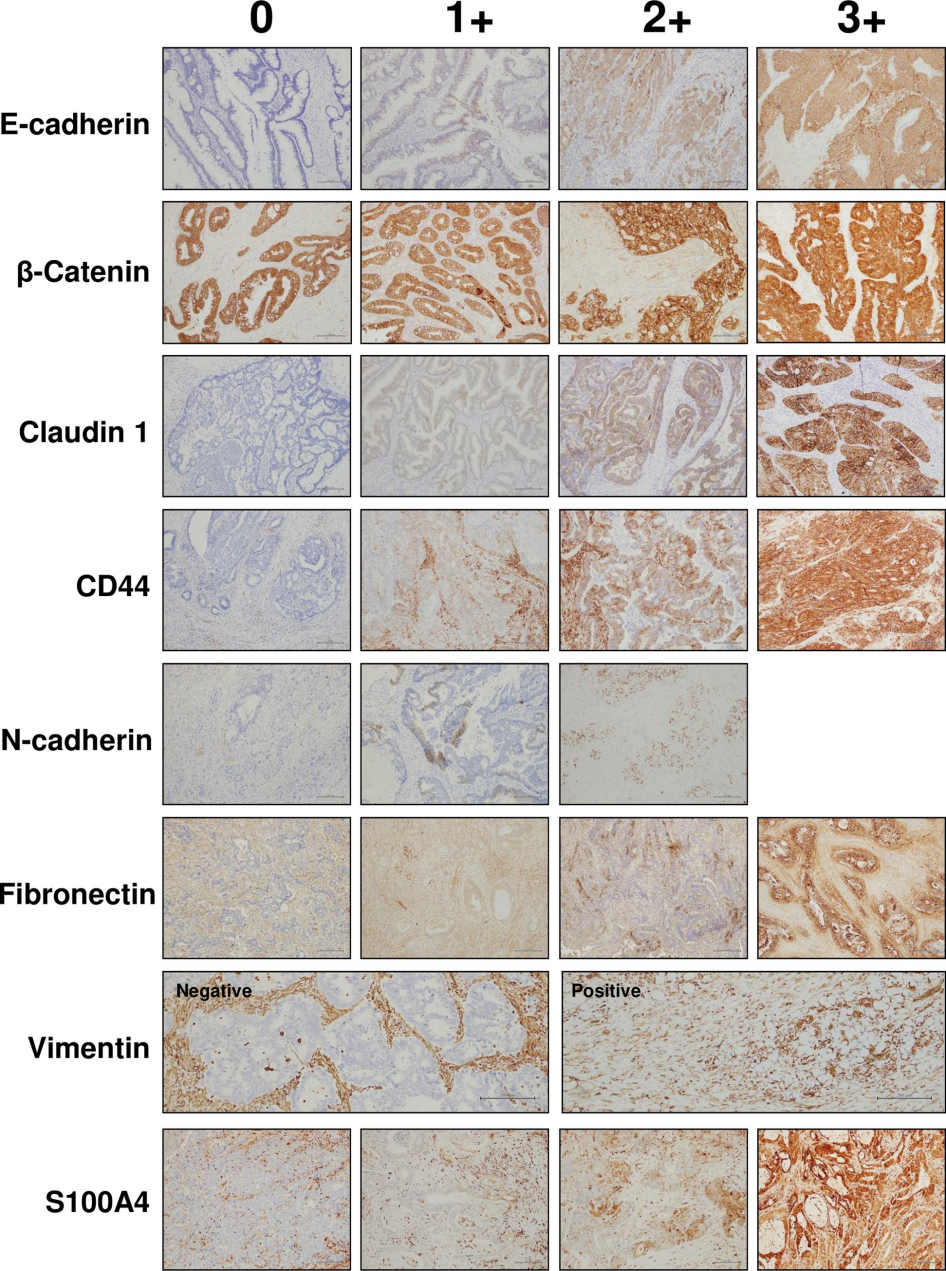
The immunohistochemical staining of EMT-related proteins. Photomicrographic examples of the semi-quantitatively scoring for the expression of eight EMT-related proteins by IHC (Original magnification 100x, Bar = 200 μm).

**Fig 2:**
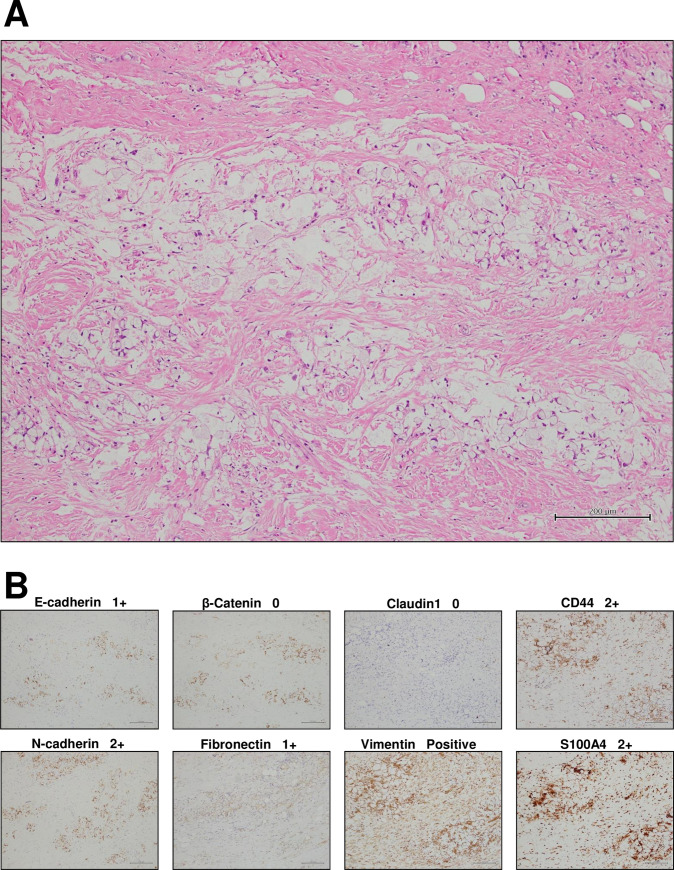
Signet-ring cell carcinoma. (A) Hematoxylin & eosin stain. (B) Immunohistochemical stain for EMT markers. (Original magnification 100x, Bar = 200 μm).

The original 150 FFPE tumoral tissue specimens were screened and eight samples were excluded, including six accepting chemo- or radio-therapy, one with no residual tumor after endoscopic resection, and one sarcoma instead of epithelial carcinoma. The clinicopathologic features of 142 samples are listed in Table 1. The expression of the eight EMT-related proteins was semi-quantitatively scored as in Table 2 and showed as examples in Fig 1. The IHC expression score of EMT-related proteins and its correlation with the histopathologic grades was statistically assessed by Chi-square test (Table 3). Increased nuclear translocation of β-catenin expression correlates with the progression of histopathologic grade (*p* < 0.001). The expression of claudin-1 decreases as the histopathologic grades progress (*p* = 0.001). Hence, decreased expression of claudin-1 and increased nuclear translocation of β-catenin are statistically associated with the progression of histopathologic grade. As to the mesenchymal markers, N-cadherin is expressed in four moderately-to-poorly differentiated cases (*p* = 0.009). Vimentin is aberrantly expressed in only one case of signet-ring cell carcinoma, which is poorly differentiated with nodal and distant metastases (*p* = 0.001) (Fig 2).

**Table 1:** Clinicopathologic data of the colorectal cancer cohort.

	Mean/Frequency	SD/Percentage
Age	66.58	13.28
Gender		
Female	54	38.03
Male	88	61.97
Tumor location		
Right colon	41	28.87
Left colon	37	26.06
Rectum	64	45.07
Stage		
0	2	1.41
Ⅰ	16	11.27
Ⅱ	41	28.87
Ⅲ	55	38.73
Ⅳ	28	19.72
Stage		
Early stage (Stage 0/Ⅰ/Ⅱ)	59	41.55
Advanced stage (Stage Ⅲ/Ⅳ)	83	58.45
Tumor extent		
Tis	2	1.41
T1	4	2.82
T2	20	14.08
T3	91	64.08
T4	25	17.61
Tumor extent		
Early (Tis/T1/T2)	26	18.31
Advanced (T3/T4)	116	81.69
Lymph node metastasis		
N0	63	44.37
N1	34	23.94
N2	45	31.69
Lymph node metastasis		
Early (N0)	63	44.37
Advanced (N1/N2)	79	55.63
Distant metastasis		
M0	113	79.58
M1	27	19.01
missing data	2	1.41
Lymphovascular invasion		
Absent	39	27.46
Present	103	72.54
Perineural invasion		
Absent	70	49.30
Present	72	50.70
Tumor deposits		
Absent	104	73.24
Present	38	26.76
N/L ratio		
<5.00	96	67.61
5.00+	45	31.69
missing data	1	0.70
CEA(ng/mL)		
<5.00	78	54.93
5.00 +	35	24.65
missing data	29	20.42
CA19-9(U/mL)		
<37.00	86	60.56
37.00 +	22	15.49
missing data	34	23.94

**Table 2:** The immunohistochemical scoring of the EMT-related proteins.

	0	1+	2+	3+
**E-cadherin**	No stain	Weak and/or faint stain	Partial and moderate membranous stain	Strong membranous stain
**β-Catenin**	Absence of nuclear stain	Nuclear stain <10%	Nuclear stain 10%-50%	Nuclear stain ≧50%
**Claudin-1**	No stain	Weak and/or faint stain	Partial and moderate membranous stain	Complete and strong membranous stain
**CD44**	No stain or weak/faint stain <10%	Partial membranous stain <30% or weak/faint stain ≧10%	Complete membranous stain <30% or partial membranous stain ≧30%	Complete membranous stain ≧30%
**N-cadherin**	No stain	Weak and/or faint stain	Partial and moderate membranous stain	Complete and strong membranous stain
**Fibronectin**	No stain or weak/faint stain <10%	Partial membranous stain <30% or weak/faint stain ≧10%	Complete membranous stain <30% or partial membranous stain ≧30%	Complete membranous stain ≧30%
**Vimentin**	No stain	Cytoplasmic stain <10%	Cytoplasmic stain 10%-50%	Cytoplasmic stain ≧50%
**S100A4**	No stain or weak/faint stain <10%	Partial membranous stain <30% or weak/faint stain ≧10%	Complete membranous stain <30% or partial membranous stain ≧30%	Complete membranous stain ≧30%

The immunohistochemical expression of the eight EMT-related proteins was semi-quantitatively scored into 0, 1+, 2+, and 3+.

**Table 3:** IHC expression of EMT-related markers *vs*. histopathologic grades.

	WD	MD	PD	*p*-value
**E-cadherin**				0.122
+1	0 (0.00)	4 (3.10)	1 (10.00)	
+2	0 (0.00)	18 (13.95)	4 (40.00)	
+3	3 (100.00)	107 (82.95)	5 (50.00)	
**β-catenin**				<0.001*
0	2 (66.67)	57 (44.53)	5 (50.00)	
+1	0 (0.00)	40 (31.25)	2 (20.00)	
+2	1 (33.33)	29 (22.66)	0 (0.00)	
+3	0 (0.00)	2 (1.56)	3 (30.00)	
**Claudin-1**				0.001*
0	0 (0.00)	8 (6.20)	4 (40.00)	
+1	3 (100.00)	42 (32.56)	4 (40.00)	
+2	0 (0.00)	58 (44.96)	2 (20.00)	
+3	0 (0.00)	21 (16.28)	0 (0.00)	
**CD44**				0.805
0	0 (0.00)	10 (7.81)	0 (0.00)	
+1	0 (0.00)	42 (32.81)	3 (30.00)	
+2	2 (66.67)	55 (42.97)	5 (50.00)	
+3	1 (33.33)	21 (16.41)	2 (20.00)	
**N-cadherin**				0.009*
0	3 (100.00)	126 (97.67)	9 (90.00)	
+1	0 (0.00)	3 (2.33)	0 (0.00)	
+2	0 (0.00)	0 (0.00)	1 (10.00)	
**Fibronectin**				0.187
0	1 (33.33)	9 (6.98)	2 (20.00)	
+1	1 (33.33)	75 (58.14)	8 (80.00)	
+2	1 (33.33)	42 (32.56)	0 (0.00)	
+3	0 (0.00)	3 (2.33)	0 (0.00)	
**Vimentin**				0.001*
negative	3 (100.00)	129 (100.00)	9 (90.00)	
positive	0 (0.00)	0 (0.00)	1 (10.00)	
**S100A4**				0.593
0	1 (33.33)	35 (27.13)	2 (20.00)	
+1	2 (67.67)	57 (44.19)	3 (30.00)	
+2	0 (0.00)	22 (17.05)	4 (40.00)	
+3	0 (0.00)	15 (11.63)	1 (10.00)	

WD: well differentiated, MD: moderately differentiated, PD: poorly differentiated.

### The correction of IHC expression of EMT-related markers and cancer stages

The expression of EMT-related proteins does not show statistically significant correlation with the cancer stages by Chi-square test (Table 4), if the cohort is classified into stages 0 to IV according the American Joint Committee on Cancer (AJCC) Cancer Staging Manual, 8^th^ Edition [15]. However, if stages 0 to II are classified as early stages and stages III to IV are classified as advanced stages (Table 5), the expression of E-cadherin is lowered in the advanced stages than that in the early stages (*p* = 0.041). Hence, decreased expression of E-cadherin correlates with the tumor progression of CRC.

**Table 4:** IHC expression of EMT-related markers *vs*. cancer stages classified into 0 to IV.

**Cancer Stage**						
	0	Ⅰ	Ⅱ	Ⅲ	Ⅳ	*p*-value
**E-cadherin**						0.201
+1	0 (0.00)	0 (0.00)	0 (0.00)	4 (7.27)	1 (3.57)	
+2	0 (0.00)	3 (18.75)	3 (7.32)	8 (14.55)	8 (28.57)	
+3	2 (100.00)	13 (81.25)	38 (92.68)	43 (78.18)	19 (67.86)	
**β-catenin**						0.951
0	1 (50.00)	9 (56.25)	17 (41.46)	22 (40.74)	15 (53.57)	
+1	1 (50.00)	5 (31.25)	14 (34.15)	15 (27.78)	7 (25.00)	
+2	0 (0.00)	2 (12.50)	8 (19.51)	15 (27.78)	5 (17.86)	
+3	0 (0.00)	0 (0.00)	2 (4.88)	2 (3.70)	1 (3.57)	
**Claudin-1**						0.293
0	1 (50.00)	1 (6.25)	0 (0.00)	7 (12.73)	3 (10.71)	
+1	1 (50.00)	7 (43.75)	15 (36.59)	16 (29.09)	10 (35.71)	
+2	0 (0.00)	7 (43.75)	19 (46.34)	25 (45.45)	9 (32.14)	
+3	0 (0.00)	1 (6.25)	7 (17.07)	7 (12.73)	6 (21.43)	
**CD44**						0.646
0	0 (0.00)	2 (12.50)	1 (2.44)	5 (9.26)	2 (7.14)	
+1	1 (50.00)	5 (31.25)	10 (24.39)	16 (29.63)	13 (46.43)	
+2	1 (50.00)	5 (31.25)	21 (51.22)	24 (44.44)	11 (39.29)	
+3	0 (0.00)	4 (25.00)	9 (21.95)	9 (16.67)	2 (7.14)	
**N-cadherin**						0.350
0	2 (100.00)	16 (100.00)	41 (100.00)	52 (94.55)	27 (96.43)	
+1	0 (0.00)	0 (0.00)	0 (0.00)	3 (5.45)	0 (0.00)	
+2	0 (0.00)	0 (0.00)	0 (0.00)	0 (0.00)	1 (3.57)	
**Fibronectin**						0.291
0	1 (50.00)	2 (12.50)	2 (4.88)	6 (10.91)	1 (3.57)	
+1	0 (0.00)	11 (68.75)	20 (48.78)	33 (60.00)	20 (71.43)	
+2	1 (50.00)	3 (18.75)	18 (43.90)	15 (27.27)	6 (21.43)	
+3	0 (0.00)	0 (0.00)	1 (2.44)	1 (1.82)	1 (3.57)	
**Vimentin**						0.393
negative	2 (100.00)	16 (100.00)	41 (100.00)	55 (100.00)	27 (96.43)	
positive	0 (0.00)	0 (0.00)	0 (0.00)	0 (0.00)	1 (3.57)	
**S100A4**						0.208
0	1 (50.00)	10 (62.50)	7 (17.07)	13 (23.64)	7 (25.00)	
+1	1 (50.00)	3 (18.75)	21 (51.22)	27 (49.90)	10 (35.71)	
+2	0 (0.00)	2 (12.50)	8 (19.51)	9 (16.36)	7 (25.00)	
+3	0 (0.00)	1 (6.25)	5 (12.20)	6 (10.91)	4 (14.29)	

**Table 5:** IHC expression of EMT-related markers *vs*. cancer stages classified into early stage (stages 0 to II) and advanced stage (stages III to IV).

**Cancer Stage**			
	Early stage	Advanced stage	*p*-value
Number	57	83	
**E-cadherin**			0.041*
+1	0 (0.00)	5 (6.02)	
+2	6 (10.17)	16 (19.28)	
+3	53 (89.83)	62 (74.70)	
**β-catenin**			0.687
0	27 (45.76)	37 (45.12)	
+1	20 (33.90)	22 (26.83)	
+2	10 (16.95)	20 (24.39)	
+3	2 (3.39)	3 (3.66)	
**Claudin-1**			0.281
0	2 (3.39)	10 (12.05)	
+1	23 (38.98)	26 (31.33)	
+2	26 (44.07)	34 (40.96)	
+3	8 (13.56)	13 (15.66)	
**CD44**			0.411
0	3 (5.08)	7 (8.54)	
+1	16 (27.12)	29 (35.37)	
+2	27 (45.76)	35 (42.68)	
+3	13 (22.03)	11 (13.41)	
**CD44**			0.160
0 or +1	19 (32.20)	36 (43.90)	
+2 or +3	40 (67.80)	46 (56.10)	
**N-cadherin**			0.227
0	59 (100.00)	78 (95.12)	
+1	0 (0.00)	3 (3.66)	
+2	0 (0.00)	1 (1.22)	
**Fibronectin**			0.481
0	5 (8.47)	7 (8.43)	
+1	31 (52.54)	53 (63.86)	
+2	22 (37.29)	21 (25.30)	
+3	1 (1.69)	2 (2.41)	
**Vimentin**			0.397
negative	59 (100.00)	82 (98.80)	
positive	0 (0.00)	1 (1.20)	
**S100A4**			0.855
0	18 (30.51)	20 (24.10)	
+1	25 (42.37)	37 (44.58)	
+2	10 (16.95)	16 (19.28)	
+3	6 (10.17)	10 (12.05)	

### The correlation of IHC expression of EMT-related markers and other clinicopathologic features

The expression of EMT-related proteins and its correlation with the other clinicopathologic features was statistically assessed by Chi-square test and *t*-test. The expression of E-cadherin decreases as the progress of regional lymph node metastasis (*p* = 0.021) (Table 6). The expression of E-cadherin and claudin-1 is lower in the presence of tumor deposits (*p* = 0.004 and 0.007, respectively) (Table 7). Among the patients with elevated serum CA 19–9 levels (≧37 U/mL), the nuclear translocation of β-catenin decreases (*p* = 0.001) (Table 8). In this study, we also found that elevated serum levels of CA 19–9 are statistically associated with prognostic pathologic features including regional lymph node metastasis (*p* = 0.006), distant metastasis (*p* = 0.012), lymphovascular invasion (*p* = 0.023), perineural invasion (*p* = 0.027), and tumor deposits (*p* = 0.005) (S1 Table). In addition, the left-sided colon and rectal cancers show increased nuclear translocation of β-catenin compared to the right-sided colon cancers (*p* = 0.017), while the rectal cancers show increased fibronectin expression compared to the right-sided and left-sided colon cancers (*p* = 0.026) (Table 9). In summary, decreased expression of the epithelial markers E-cadherin, claudin-1, and β-catenin nuclear location are statistically associated with other important prognostic factors, including nodal metastasis, tumor deposits, and elevated serum CA 19–9 levels.

**Table 6:** IHC expression of EMT-related markers *vs*. regional lymph node metastasis.

**Regional Lymph Node Matastasis**				
	N0	N1	N2	*p*-value
Number	63	34	45	
**E-cadherin**				0.021^*^
+1	0 (0.00)	1 (2.94)	4 (8.89)	
+2	6 (9.52)	5 (14.71)	11 (24.44)	
+3	57 (90.48)	28 (82.35)	30 (66.67)	
**β-catenin**				0.365
0	30 (47.62)	13 (38.24)	21 (47.73)	
+1	21 (33.33)	12 (35.29)	9 (20.45)	
+2	10 (15.87)	9 (26.47)	11 (25.00)	
+3	2 (3.17)	0 (0.00)	3 (6.82)	
**Claudin-1**				0.367
0	2 (3.17)	3 (8.82)	7 (15.56)	
+1	24 (38.10)	10 (29.41)	15 (33.33)	
+2	27 (42.86)	17 (50.00)	16 (35.56)	
+3	10 (15.87)	4 (11.76)	7 (15.56)	
**CD44**				0.502
0	3 (4.76)	3 (8.82)	4 (9.02)	
+1	18 (28.57)	9 (26.47)	18 (40.91)	
+2	28 (44.44)	16 (47.06)	18 (40.91)	
+3	14 (22.22)	6 (17.65)	4 (9.09)	
**CD44**				0.194
0 or +1	21 (33.33)	12 (35.29)	22 (50.00)	
+2 or +3	42 (66.67)	22 (64.71)	22 (50.00)	
**N-cadherin**				0.214
0	62 (100.00)	32 (94.12)	43 (95.56)	
+1	0 (0.00)	2 (5.88)	1 (2.22)	
+2	0 (0.00)	0 (0.00)	1 (2.22)	
**Fibronectin**				0.597
0	5 (7.94)	3 (8.82)	4 (8.89)	
+1	35 (55.56)	21 (61.67)	28 (62.22)	
+2	22 (34.92)	8 (23.53)	13 (28.89)	
+3	1 (5.59)	2 (5.88)	0 (0.00)	
**Vimentin**				0.338
negative	63 (100.00)	34 (100.00)	44 (97.78)	
positive	0 (0.00)	0 (0.00)	1 (2.22)	
**S100A4**				0.880
0	19 (30.16)	7 (20.59)	12 (26.67)	
+1	26 (41.27)	17 (50.00)	19 (42.22)	
+2	11 (17.46)	5 (14.71)	10 (22.22)	
+3	7 (11.11)	5 (14.71)	4 (8.89)	

**Table 7:** IHC expression of EMT-related markers *vs*. tumor deposits.

**Tumor Deposits**				
		Absent	Present	*p*-value
Number		104	38	
**E-cadherin**				0.004*
	+1	1 (0.96)	4 (10.53)	
	+2	13 (12.50)	9 (23.68)	
	+3	90 (86.54)	25 (65.79)	
**β-catenin**				0.736
	0	46 (44.66)	18 (47.37)	
	+1	33 (32.04)	9 (23.68)	
	+2	21 (20.39)	9 (23.68)	
	+3	3 (2.91)	2 (5.26)	
**Claudin-1**				0.007*
	0	4 (3.85)	8 (21.05)	
	+1	40 (38.46)	9 (23.68)	
	+2	43 (41.35)	17 (44.74)	
	+3	17 (16.35)	4 (10.53)	
**CD44**				0.346
	0	5 (4.85)	5 (13.16)	
	+1	35 (33.98)	10 (26.32)	
	+2	46 (44.66)	16 (42.11)	
	+3	17 (16.50)	7 (18.42)	
**CD44**				1.000
	0 or +1	40 (38.83)	15 (39.47)	
	+2 or +3	63 (61.17)	23 (60.53)	
**N-cadherin**				0.072
	0	102 (99.03)	35 (92.11)	
	+1	1 (0.97)	2 (5.26)	
	+2	0 (0.00)	1 (2.63)	
**Fibronectin**				0.196
	0	6 (5.77)	6 (15.79)	
	+1	62 (59.62)	22 (57.89)	
	+2	33 (31.73)	10 (26.32)	
	+3	3 (2.88)	0 (0.00)	
**Vimentin**				0.268
	negative	104 (100.00)	37 (97.37)	
	positive	0 (0.00)	1 (2.63)	
**S100A4**				0.384
	0	24 (23.08)	14 (36.84)	
	+1	48 (46.15)	14 (36.84)	
	+2	19 (18.27)	7 (18.42)	
+3		13 (12.50)	3 (7.89)	

**Table 8:** IHC expression of EMT-related markers *vs*. serum levels of CA 19–9.

Carbohydrate Antigen 19–9			
	<37.00	37.00 +	*p*-value
Number	86	22	
**E-cadherin**			0.293
+1	3 (3.49)	1 (4.55)	
+2	9 (10.47)	5 (22.73)	
+3	74 (86.05)	16 (72.73)	
**β-catenin**			0.01*
0	30 (35.29)	16 (72.73)	
+1	27 (31.76)	5 (22.73)	
+2	23 (27.06)	1 (4.55)	
+3	5 (5.88)	0 (0.00)	
**Claudin-1**			0.148
0	5 (5.81)	3 (13.64)	
+1	32 (37.21)	5 (22.73)	
+2	31 (36.05)	12 (54.55)	
+3	18 (20.93)	2 (9.09)	
**CD44**			0.621
0	6 (7.06)	0 (0.00)	
+1	29 (34.12)	8 (36.36)	
+2	38 (44.71)	10 (45.45)	
+3	12 (14.12)	4 (18.18)	
**CD44**			0.682
0 or +1	35 (41.18)	8 (36.36)	
+2 or+3	50 (58.82)	14 (63.64)	
**N-cadherin**			1.000
0	82 (96.47)	22 (100.00)	
+1	3 (3.53)	0 (0.00)	
**Fibronectin**			0.141
0	9 (10.47)	1 (4.55)	
+1	45 (52.33)	17 (77.27)	
+2	30 (34.88)	11 (31.43)	
+3	2 (2.33)	1 (4.55)	
**Vimentin**			1.000
negative	86 (100.00)	22 (100.00)	
**S100A4**			0.917
0	24 (27.91)	7 (31.82)	
+1	35 (40.70)	9 (40.91)	
+2	17 (19.77)	3 (13.64)	
+3	10 (11.63)	3 (13.64)	

**Table 9:** IHC expression of EMT-related markers *vs*. primary tumor locations.

**Primary Tumor Location**				
	Right colon	Left colon	Rectum	*p*-value
**E-cadherin**				0.464
+1	1 (2.44)	0 (0.00)	4 (6.25)	
+2	8 (19.51)	5 (13.51)	9 (14.06)	
+3	32 (78.05)	32 (86.49)	51 (79.69)	
**β-catenin**				0.017*
0	29 (70.73)	13 (35.14)	22 (34.92)	
+1	6 (14.63)	13 (34.14)	23 (36.51)	
+2	5 (12.50)	10 (27.03)	15 (23.81)	
+3	1 (2.44)	1 (2.70)	3 (4.76)	
**Claudin-1**				0.603
0	4 (9.76)	3 (8.11)	5 (7.81)	
+1	16 (39.02)	16 (43.24)	17 (26.56)	
+2	17 (41.46)	13 (35.14)	30 (46.88)	
+3	4 (9.76)	5 (13.51)	12 (18.75)	
**CD44**				0.122
0	1 (2.44)	3 (8.11)	6 (9.52)	
+1	10 (24.39)	15 (40.54)	20 (31.75)	
+2	18 (43.90)	17 (45.95)	27 (42.86)	
+3	12 (29.27)	2 (5.41)	10 (15.87)	
**CD44**				0.126
0 or +1	11 (26.83)	18 (48.65)	26 (41.27)	
+2 or +3	30 (73.17)	19 (51.3)	37 (58.73)	
**N-cadherin**				0.187
0	40 (97.56)	37 (100.00)	61 (95.31)	
+1	0 (0.00)	0 (0.00)	3 (4.69)	
+2	1 (2.44)	0 (0.00)	0 (0.00)	
**Fibronectin**				0.026*
0	8 (19.51)	0 (0.00)	4 (6.25)	
+1	23 (56.10)	23 (62.16)	38 (59.38)	
+2	10 (24.39)	14 (37.84)	19 (29.69)	
+3	0 (0.00)	0 (0.00)	3 (4.69)	
**Vimentin**				0.289
negative	40 (97.56)	37 (100.00)	64 (100.00)	
positive	1 (2.44)	0 (0.00)	0 (0.00)	
**S100A4**				0.167
0	10 (24.39)	8 (21.62)	20 (31.25)	
+1	24 (58.54)	15 (40.54)	23 (35.94)	
+2	6 (14.63)	9 (24.32)	11 (17.19)	
+3	1 (2.44)	5 (13.51)	10 (15.63)	

### Methylation levels of DNA CpG sites in *CD44* gene promoter

Bisulfite conversion of the extracted genomic DNA with subsequent PCR and NGS was performed for comparison of the DNA CpG methylation status of *CD44* gene between the tumoral and non-tumoral freshly frozen tissues. The 80 sequencing data were deposited in NCBI Sequence Read Archive (accession number: PRJNA736199). Among the 27 CpG sites, the CpG methylation ratios are low (< 5%) except site -385 (about 45%) and site +39 (about 56%). Using the *t*-test analysis, the DNA methylation levels of *CD44* gene promoter between the tumoral and non-tumoral tissues showed statistical difference (*p* < 0.05) on six CpG sites, including -310 bp, -301 bp, -253 bp, -217 bp, -68 bp, and +16 bp from the transcription starting site (Table 10). In spite of these differences are significant, the methylation ratios of 6 CpG sites are too low to affect gene expression.

**Table 10:** Methylation levels of DNA CpG sites in *CD44* gene promoter between (A) 50 tumoral *vs*. non-tumoral tissues; (B) 30 paired tumoral *vs*. non-tumoral tissues.

(A)			
CpG site position (TSS+1)	Methylation ratio (%) in tumor (n = 50)	Methylation ratio (%) in non-tumor (n = 30)	*p*-value
-385	46.57 ± 7.9	45.73 ± 7.78	0.643
-339	0.95 ± 0.26	0.92 ± 0.13	0.456
-334	1.26 ± 0.37	1.15 ± 0.19	0.083
-315	1.42 ± 0.35	1.39 ± 0.19	0.611
-312	2.08 ± 0.51	2.10 ± 0.21	0.832
-310	0.93 ± 0.37	0.72 ± 0.15	0.001*
-301	1.32 ± 0.57	1.09 ± 0.17	0.012*
-282	1.18 ± 0.29	1.20 ± 0.16	0.640
-272	1.62 ± 0.27	1.61 ± 1.16	0.828
-253	1.45 ± 0.30	1.60 ± 0.20	0.008*
-236	0.89 ± 0.18	0.89 ± 0.12	0.982
-234	0.90 ± 0.19	0.88 ± 0.11	0.576
-217	0.85 ± 0.21	0.94 ± 0.13	0.032*
-210	0.69 ± 0.14	0.70 ± 0.12	0.697
-195	1.13 ± 0.39	1.15 ± 0.23	0.814
-169	1.06 ± 0.22	1.04 ± 0.23	0.800
-164	0.79 ± 0.17	0.84 ± 0.10	0.134
-148	0.97 ± 0.25	0.98 ± 0.16	0.821
-144	1.23 ± 0.53	1.08 ± 0.21	0.088
-114	0.26 ± 0.09	0.27 ± 0.05	0.473
-72	0.50 ± 0.17	0.46 ± 0.13	0.348
-68	0.60 ± 0.14	0.58 ± 0.10	0.505
-55	0.50 ± 0.17	0.49 ± 0.09	0.943
-30	0.50 ± 0.15	0.55 ± 0.10	0.115
-6	0.42 ± 0.12	0.40 ± 0.06	0.281
+16	0.43 ± 0.18	0.50 ± 0.14	0.046*
+39	57.98 ± 4.63	56.79 ± 3.90	0.244
(B)			
CpG site position (TSS+1)	Methylation ratio (%) in tumor (n = 30)	Methylation ratio (%) in non-tumor (n = 30)	*p*-value
-385	45.84 ± 7.72	45.73 ± 7.78	0.955
-339	0.92 ± 0.13	0.92 ± 0.13	0.849
-334	1.21 ± 0.23	1.15 ± 0.19	0.277
-315	1.42 ± 0.15	1.39 ± 0.19	0.463
-312	1.99 ± 0.27	2.10 ± 0.21	0.097
-310	0.75 ± 0.16	0.72 ± 0.15	0.501
-301	1.09 ± 0.18	1.09 ± 0.17	0.951
-282	1.20 ± 0.12	1.20 ± 0.16	0.985
-272	1.60 ± 0.15	1.61 ± 0.16	0.894
-253	1.60 ± 0.18	1.60 ± 0.20	0.925
-236	0.89 ± 0.09	0.89 ± 0.12	0.936
-234	0.88 ± 0.14	0.88 ± 0.11	0.784
-217	0.97 ± 0.13	0.94 ± 0.13	0.284
-210	0.74 ± 0.09	0.70 ± 0.12	0.168
-195	1.18 ± 0.22	1.15 ± 0.23	0.658
-169	1.03 ± 0.13	1.04 ± 0.23	0.844
-164	0.83 ± 0.12	0.84 ± 0.10	0.908
-148	1.03 ± 0.14	0.98 ± 0.16	0.219
-144	1.18 ± 0.25	1.08 ± 0.21	0.122
-114	0.29 ± 0.07	0.27 ± 0.05	0.411
-72	0.50 ± 0.10	0.46 ± 0.13	0.224
-68	0.64 ± 0.09	0.58 ± 0.10	0.033*
-55	0.51 ± 0.10	0.49 ± 0.09	0.454
-30	0.53 ± 0.11	0.55 ± 0.10	0.431
-6	0.40 ± 0.07	0.40 ± 0.06	0.968
+16	0.51 ± 0.16	0.50 ± 0.14	0.919
+39	56.89 ± 3.67	56.79 ± 3.90	0.923

## Discussion

The present study investigated the correlation of expression of 8 EMT-related genes (E-cadherin, β-catenin, claudin-1, CD44, N-cadherin, fibronectin, vimentin, S100A4) with the clinicopathologic features of CRC in Taiwan. Furthermore, CD44 expression and its DNA CpG epigenetic regulation was also studied.

Our immunohistochemical assessment showed that the expression of epithelial markers E-cadherin decreases as the progress of cancer stage (Table 5) and regional lymph node metastasis (Table 6). Loss of E-cadherin expression was reported to be significantly associated with infiltrative tumor growth pattern and advanced cancer stage, independent of other clinical, pathological and molecular features of CRC [8]. These findings suggest that E-cadherin expression may serve as a predictive marker for tumor invasion and lymph node metastasis in CRC.

The expression of claudin-1 at the mRNA and protein levels was reported to increase in the CRC tissue in comparison to that in the normal tissue specimens [16]. A recent research revealed that claudin-1 in CRC shows lowered membranous expression and, in contrast, significant cytoplasmic expression [17]. We evaluated the immunohistochemical scores of claudin-1 by its membranous expression. The expression of claudin-1 decreases as the histopathologic grades progress (Table 3) and in the presence of tumor deposits (Table 7). Our results suggest that membranous expression of claudin-1 is a marker for progress of histopathologic grade in CRC.

The nuclear expression of β-catenin increases with the progression of histopathologic grade (Table 3), but is not in accordance with lymph node metastasis (Table 6). The increase of nuclear translocation of β-catenin as cancer progressing may be associated with the Wnt/β-catenin signaling pathway [18]. Wnt signal blocks the ubiquitination of β-catenin by β-transducing repeat-containing protein (β-TrCP). Newly synthesized β-catenin accumulates, then enters the nucleus and drives target gene expression. In a multivariate study, lymph node metastasis and high expression of nuclear β-catenin are independent prognostic factors for patient survival of CRC [19]. A research investigating the prognostic and diagnostic significance of β-catenin immunostaining suggested that the occurrence of nuclear β-catenin correlated with the sequential stages in colorectal carcinogenesis, and high immunohistochemical scores in CRC were significantly associated with lymph node metastasis and poor survival [20]. Our data, however, shows no correlation between nuclear β-catenin and lymph node metastasis (Table 6). The nuclear expression of β-catenin only indicated progressive histopathologic differentiation of CRC in the present study (Table 3).

In our immunohistochemical analysis, vimentin expression is negative in most of the CRC specimens, but is aberrantly expressed in one case of signet-ring cell carcinoma (Fig 2), but in one comprehensive immunohistochemical research of primary signet-ring cell carcinoma in the stomach and colorectum from Japan [21], none of the 42 signet-ring cell carcinomas (30 gastric and 12 colorectal) express vimentin. Aberrant methylation of the *VIM* gene is common (65%) in CRC [11]. The hypermethylation of *VIM* gene might cause negative IHC staining in our study. The relationship between vimentin expression and *VIM* methylation of CRC may need further investigation.

Histology and molecular features of right-sided (proximal) colon cancers are different when compared with left-sided (distal) colon cancers and rectal cancers [22]. In our immunohistochemical assessment, the left-sided colon and rectal cancers show increased nuclear translocation of β-catenin compared to the right colon cancers, while the rectal cancers show increased fibronectin expression compared to the right-sided and left-sided colon cancers (Table 9). This phenomenon may reflect the embryonic and molecular genetic differences in the sidedness of colorectum. Besides, left-sided CRC patients tend to have chromosomal instability-high (CIN-high) tumors [22]. The high β-catenin nuclear translocation and fibronectin expression of rectal cancers possibly provide some pathological and therapeutic clues.

The DNA methylation levels of *CD44* gene promoter between the tumoral and non-tumoral tissues by NGS comparison showed statistical difference on six CpG sites, including -310 bp, -301 bp, -253 bp, -217bp, -68 bp, and +16 bp from the transcription start site (Table 10). However, such differences may not be sufficient to affect gene expression because these DNA methylation ratios are too low to inactivate gene. This is further supported by the absent of significant correlation between the tumoral CD44 expression and the clinicopathologic characteristics in our immunohistochemical assessment (Tables 3–8). The expression of CD44 may be regulated by other epigenetic or posttranslational mechanisms. A recent research in urothelial cancer showed that overexpression of *c-Myc* upregulates *CD44* through a *miR-34a*-mediated competing endogenous RNA mechanism [23]. Up to now, the role of CD44 and its variants in EMT is still controversial. The positive expression of CD44 and its variants may play important roles in the tumor infiltration, metastasis and poor prognosis of CRC [24, 25]. In contrast, some studies found low expression of CD44 or its variants was correlated with increased tumor recurrence, short disease-free survival and tumor progression [26, 27]. Furthermore, one study considers that changes in the expression level of CD44v6 mRNA do not predict tumor spread or patient survival in CRC [28]. CD44 is a transmembrane glycoprotein with many variants by alternative mRNA splicing mechanisms. One of the variants, CD44v6, engages in a variety of biological processes, including cell growth, apoptosis, migration, and angiogenesis [29]. Overexpression of CD44v6 can induce chemoresistance of 5-fluorouracil and oxaliplatin in a colon cancer cell line SW480 [30]. In sum, the dual properties of CD44 function in tumorigenesis are complicated, which may be caused by the presence of various variants and lack of immunohistochemical antibody specificity for CD44v6. In this study, although the DNA methylation levels of *CD44* gene are not significantly varied between the tumoral and non-tumoral tissues of CRC, the potential of CD44v6 as a biomarker in CRC progression should be further verified through applying a specific immunohistochemical antibody against CD44v6.

## Conclusion

This is the first study to analyze various EMT markers of CRC tissue specimens in Taiwan as far as we know. In IHC results, the decreased expression of claudin-1 as well as increased nuclear translocation of β-catenin and N-cadherin expression is statistically associated with the progression of histopathologic grade (Table 3). In addition, decreased expression of E-cadherin correlates with the progression of cancer stage (Table 5). Therefore, along with the tumor progression of CRC, the expression of epithelial markers E-cadherin and claudin-1 decreases, while the expression of nuclear β-catenin increases, indicating the close relationship of E-cadherin, claudin-1, and nuclear β-catenin expression with the tumor progression and important clinicopathologic determinants of CRC in Taiwan. The results may provide the information for prognosis evaluation and treatment prediction as well as the potentials of these markers as theranostic and prognostic markers of CRC. On the other hand, *CD44* expression and its DNA epigenetic regulation showed no correlation with clinicopathologic features of CRC in Taiwan.

## Supporting information

S1 Table The correlation of CA19-9 serum level and prognostic features. (XLSX)

## Abbreviations

CRC: colorectal cancer
EMT: epithelial-mesenchymal transition
FFPE: formalin-fixed and paraffin-embedded
IHC: immunohistochemistry
NGS: next-generation sequencing
CLDN1: claudin-1
CDH1: E-cadherin
VIM: vimentin
AJCC: American Joint Committee on Cancer

## References

[1] Bray F, Ferlay J, Soerjomataram I, Siegel RL, Torre LA, Jemal A. Global cancer statistics 2018: GLOBOCAN estimates of incidence and mortality worldwide for 36 cancers in 185 countries. CA Cancer J Clin. 2018;68(6):394–424. doi: 10.3322/caac.21492 30207593

[2] Health Promotion Administration, Ministry of Health and Welfare, Taiwan. Cancer Registry Annual Report, 2018 Taiwan. Retrieved from https://www.hpa.gov.tw/Pages/ashx/File.ashx?FilePath=~/File/Attach/13498/File_15611.pdf (Accessed on Dec. 30, 2020.)

[3] Ministry of Health and Welfare, Taiwan. Statistics on Causes of Death in Taiwan, 2019. Retrieved from https://www.mohw.gov.tw/dl-61912-c189168f-f107-4a4b-9b10-b18789a2a4e9.html (Accessed on Dec. 30, 2020.)

[4] Dekker E, Tanis PJ, Vleugels JLA, Kasi PM, Wallace MB. Colorectal cancer. Lancet.2019;394(10207):1467–80. doi: 10.1016/S0140-6736(19)32319-0 31631858

[5] Mittal V. Epithelial mesenchymal transition in tumor metastasis. Annu Rev Pathol. 2018;13:395–412. doi: 10.1146/annurev-pathol-020117-043854 29414248

[6] Kalluri R, Weinberg RA. The basics of epithelial-mesenchymal transition. J Clin Invest. 2009;119(6):1420–8. doi: 10.1172/JCI39104 PMC2689101 19487818 PMC2689101

[7] Heerboth S, Housman G, Leary M, Longacre M, Byler S, Lapinska K, et al. EMT and tumor metastasis. Clin Transl Med. 2015;4:6. doi: 10.1186/s40169-015-0048-3 PMC4385028 25852822 PMC4385028

[8] Kim SA, Inamura K, Yamauchi M, Nishihara R, Mima K, Sukawa Y, et al. Loss of CDH1 (E-cadherin) expression is associated with infiltrative tumour growth and lymph node metastasis. Br J Cancer. 2016;114(2):199–206. doi: 10.1038/bjc.2015.347 PMC4815802 26742007 PMC4815802

[9] Toiyama Y, Yasuda H, Saigusa S, Tanaka K, Inoue Y, Goel A, et al. Increased expression of Slug and Vimentin as novel predictive biomarkers for lymph node metastasis and poor prognosis in colorectal cancer. Carcinogenesis. 2013;34(11):2548–57. doi: 10.1093/carcin/bgt282 24001454

[10] Michailidi C, Theocharis S, Tsourouflis G, Pletsa V, Kouraklis G, Patsouris E, et al. Expression and promoter methylation status of hMLH1, MGMT, APC, and CDH1 genes in patients with colon adenocarcinoma. Exp Biol Med (Maywood). 2015;240(12):1599–605. doi: 10.1177/1535370215583800 PMC4935349 25908636 PMC4935349

[11] Shirahata A, Sakata M, Sakuraba K, Goto T, Mizukami H, Saito M, et al. Vimentin methylation as a marker for advanced colorectal carcinoma. Anticancer Res. 2009;29(1):279–81. 19331162

[12] Verkaik NS, van Steenbrugge GJ, van Weerden WM, Bussemakers MJ, van der Kwast TH. Silencing of CD44 expression in prostate cancer by hypermethylation of the CD44 promoter region. Lab Invest. 2000;80:1291–8. doi: 10.1038/labinvest.3780137 10950120

[13] Woodson K, O’Reilly KJ, Ward DE, Walter J, Hanson J, Walk EL, et al. CD44 and PTGS2 methylation are independent prognostic markers for biochemical recurrence among prostate cancer patients with clinically localized disease. Epigenetics. 2006;1(4):183–6. doi: 10.4161/epi.1.4.3530 17998819

[14] Sato S, Yokozaki H, Yasui W, Nikai H, Tahara E. Silencing of the CD44 gene by CpG methylation in a human gastric carcinoma cell line. Jpn J Cancer Res. 1999;90(5):485–9. doi: 10.1111/j.1349-7006.1999.tb00773.x PMC5926100 10391086 PMC5926100

[15] Wang Z, Tang Y, Xie L, Huang A, Xue C, Gu Z, et al. The prognostic and clinical value of CD44 in colorectal cancer: A meta-analysis. Front Oncol. 2019;9:309. doi: 10.3389/fonc.2019.00309 PMC6503057 31114754 PMC6503057

[16] Huo Q, Kinugasa T, Wang L, Huang J, Zhao J, Shibaguchi H, et al. Claudin-1 protein is a major factor involved in the tumorigenesis of colorectal cancer. Anticancer Res. 2009;29(3):851–7. 19414319

[17] Hahn-Stromberg V, Askari S, Ahmad A, Befekadu R, Nilsson TK. Expression of claudin 1, claudin 4, and claudin 7 in colorectal cancer and its relation with CLDN DNA methylation patterns. Tumour Biol. 2017;39(4):1010428317697569. doi: 10.1177/1010428317697569 28381183

[18] Clevers H, Nusse R. Wnt/beta-catenin signaling and disease. Cell. 2012;149(6):1192–205. doi: 10.1016/j.cell.2012.05.012 22682243

[19] Yoshida N, Kinugasa T, Ohshima K, Yuge K, Ohchi T, Fujino S, et al. Analysis of Wnt and beta-catenin expression in advanced colorectal cancer. Anticancer Res. 2015;35(8):4403–10. 26168479

[20] Wong SC, Lo ES, Lee KC, Chan JK, Hsiao WL. Prognostic and diagnostic significance of beta-catenin nuclear immunostaining in colorectal cancer. Clin Cancer Res. 2004;10(4):1401–8. doi: 10.1158/1078-0432.ccr-0157-03 14977843

[21] Terada T. An immunohistochemical study of primary signet-ring cell carcinoma of the stomach and colorectum: III. Expressions of EMA, CEA, CA19-9, CDX-2, p53, Ki-67 antigen, TTF-1, vimentin, and p63 in normal mucosa and in 42 cases. Int J Clin Exp Pathol. 2013;6(4):630–8. PMC3606852 23573309 PMC3606852

[22] Baran B, Mert Ozupek N, Yerli Tetik N, Acar E, Bekcioglu O, Baskin Y. Difference between left-sided and right-sided colorectal cancer: A focused review of literature. Gastroenterology Res. 2018;11(4):264–73. doi: 10.14740/gr1062w PMC6089587 30116425 PMC6089587

[23] Chen PC, Yu CC, Huang WY, Huang WH, Chuang YM, Lin RI, et al. c-Myc acts as a competing endogenous RNA to sponge miR-34a, in the upregulation of CD44, in urothelial carcinoma. Cancers.2019;11(10):1457. doi: 10.3390/cancers11101457 31569404 PMC6826510

[24] Li XD, Ji M, Wu J, Jiang JT, Wu CP. Clinical significance of CD44 variants expression in colorectal cancer. Tumori. 2013;99(1):88–92. doi: 10.1700/1248.13794 23549006

[25] Wu Q, Yang Y, Wu S, Li W, Zhang N, Dong X, et al. Evaluation of the correlation of KAI1/CD82, CD44, MMP7 and beta-catenin in the prediction of prognosis and metastasis in colorectal carcinoma. Diagn Pathol. 2015;10:176. doi: 10.1186/s13000-015-0411-0 PMC4582888 26408312 PMC4582888

[26] Hong I, Hong SW, Chang YG, Lee WY, Lee B, Kang YK, et al. Expression of the cancer stem cell markers CD44 and CD133 in colorectal cancer: An immunohistochemical staining analysis. Ann Coloproctol. 2015;31(3):84–91. doi: 10.3393/ac.2015.31.3.84 PMC4496458 26161375 PMC4496458

[27] Lugli A, Iezzi G, Hostettler I, Muraro MG, Mele V, Tornillo L, et al. Prognostic impact of the expression of putative cancer stem cell markers CD133, CD166, CD44s, EpCAM, and ALDH1 in colorectal cancer. Br J Cancer. 2010;103(3):382–90. doi: 10.1038/sj.bjc.6605762 PMC2920016 20606680 PMC2920016

[28] Jungling B, Menges M, Goebel R, Wittig BM, Weg-Remers S, Pistorius G, et al. Expression of CD44v6 has no prognostic value in patients with colorectal cancer. Z Gastroenterol. 2002;40(4):229–33. doi: 10.1055/s-2002-25152 11961731

[29] Ma L, Dong L, Chang P. CD44v6 engages in colorectal cancer progression. Cell Death Dis. 2019;10:30, doi: 10.1038/s41419-018-1265-7 30631039 PMC6328617

[30] Lv L, Liu HG, Dong SY, Yang F, Wang QX, Guo GL, et al. Upregulation of CD44v6 contributes to acquired chemoresistance via the modulation of autophagy in colon cancer SW480 cells. Tumour Biol. 2016;37(7):8811–24. doi: 10.1007/s13277-015-4755-6 26747179

